# RNF39 promotes colorectal cancer progression by driving RINT1 degradation and suppressing ER stress‐induced apoptosis

**DOI:** 10.1002/ctm2.70577

**Published:** 2025-12-28

**Authors:** Lu Chen, Chunluan Yuan, Teng Yu, Kaiyuan Hui, Xiuming Li, Xiaozhu Shen, Xiaodong Jiang, Bin Liu

**Affiliations:** ^1^ Jiangsu Key Laboratory of Marine Pharmaceutical Compound Screening, College of Pharmacy Jiangsu Ocean University Lianyungang China; ^2^ Department of Oncology Lianyungang Clinical College of Nanjing Medical University/The First People's Hospital of Lianyungang Lianyungang China; ^3^ Department of Oncology The Affiliated Lianyungang Hospital of Xuzhou Medical University/The First People's Hospital of Lianyungang Lianyungang China; ^4^ Department of Pathology Shanghai Ruijin Hospital Shanghai Jiao Tong University School of Medicine Shanghai China; ^5^ Department of Geriatrics Lianyungang Second People's Hospital Lianyungang China

**Keywords:** COAD, ER stress, RINT1, RNF39, ubiquitin‐dependent degradation

## Abstract

**Background:**

Colorectal adenocarcinoma (COAD) cells exploit stress‐adaptation programs, such as the unfolded protein response (UPR), to survive in hostile tumour microenvironments. However, the role of specific E3 ubiquitin ligases in regulating these survival pathways remains poorly understood. We investigated Ring Finger Protein 39 (RNF39), an E3 ligase previously implicated in immune signalling, as a potential regulator of COAD progression.

**Methods:**

We analyzed RNF39 expression using public transcriptomic datasets (TCGA, GEO) and a clinical COAD cohort via immunohistochemistry. Functional roles were assessed in COAD cell lines using shRNA knockdown, CRISPR/Cas9 knockout, and overexpression systems. In vitro assays (proliferation, invasion, colony formation) and in vivo xenograft models were employed. Mechanistic investigations included co‐immunoprecipitation, ubiquitination assays, chromatin immunoprecipitation, and luciferase reporter assays to delineate the MEF2D‐RNF39‐RINT1 axis.

**Results:**

RNF39 was aberrantly upregulated in COAD tissues, and its high expression correlated with poor patient survival. We identified the transcription factor MEF2D as a direct activator of RNF39. Functionally, RNF39 promoted COAD cell proliferation and invasion in vitro and tumour growth in vivo, dependent on its E3 ligase activity. Mechanistically, RNF39 directly interacted with, polyubiquitinated (K48‐linked), and promoted the proteasomal degradation of RAD50‐interacting protein 1 (RINT1). Consequently, RNF39 depletion stabilized RINT1, amplified the UPR and CHOP expression, and sensitized cells to ER stress‐induced apoptosis. Crucially, the anti‐tumour phenotypes of RNF39 loss were partially reversed by simultaneous RINT1 knockdown.

**Conclusion:**

RNF39 acts as a pro‐tumorigenic E3 ligase in COAD by driving the degradation of RINT1, thereby suppressing ER stress‐induced apoptosis and promoting malignant progression. Our findings delineate a novel MEF2D‐RNF39‐RINT1 signalling axis that governs tumour cell adaptation to stress. Targeting RNF39 could represent a promising therapeutic strategy to overcome stress resistance in COAD.

**Key points:**

- RNF39 is identified as an oncogenic E3 ubiquitin ligase that is upregulated in colorectal adenocarcinoma and associated with poor prognosis.
- Mechanistically, RNF39 targets the tumour suppressor RINT1 for K48‐linked polyubiquitination and proteasomal degradation.
- By degrading RINT1, RNF39 suppresses the unfolded protein response (UPR) and limits endoplasmic reticulum (ER) stress‐induced apoptosis, thereby promoting tumour progression.
- The study reveals a novel MEF2D‐RNF39‐RINT1 axis governing stress adaptation, positioning RNF39 as a potential prognostic biomarker and therapeutic target in colorectal cancer.

## Graphical abstract

RNF39, an E3 ubiquitin ligase upregulated by MEF2D in COAD, promotes RINT1 degradation, affecting UPR and ER stress. Loss of RNF39 leads to RINT1 accumulation, enhancing CHOP expression and sensitizing cells to apoptosis.

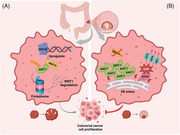

## 1 INTRODUCTION

Colorectal adenocarcinoma (COAD) remains a major clinical challenge. Although early detection and modern treatments have improved outcomes for some patients, those with advanced or metastatic disease still face unfavourable prognoses because of intratumoral heterogeneity and resistance to therapy.^1^, ^2^, ^3^ Defining how molecular networks coordinate tumour progression, stress adaptation and cell‐fate control is critical to advancing diagnostic and therapeutic development.

The endoplasmic reticulum (ER) is central to proteostasis, regulation of intracellular Ca^2^⁺ and detection of stress signals.^4^, ^5^ Disturbance of ER homeostasis − provoked by hypoxia, nutrient deprivation, oxidative stress or oncogenic signalling − promotes the buildup of misfolded proteins and activates the unfolded protein response (UPR).^6^ Although a transient UPR is adaptive and restores proteostasis, persistent or unresolved ER stress activates the PERK–eIF2α–ATF4–CHOP axis and ultimately triggers caspase‐3/7–executed apoptosis.^7^, ^8^ In colorectal cancer, increasing evidence indicates that the ability of tumour cells to endure or evade ER stress–induced apoptosis critically shapes disease progression, chemoresistance and metastatic potential.^9^, ^10^ How cancer cells reprogramme the UPR to sustain survival remains only partially defined.

The RING finger protein (RNF) family comprises a broad group of E3 ubiquitin ligases characterized by a conserved RING domain that mediates substrate‐specific ubiquitination.^11^ These enzymes regulate diverse biological processes by targeting proteins for proteasomal degradation and maintaining proteostasis. Several RNF proteins have established roles in tumour biology: RNF43 negatively regulates Wnt signalling and acts as a tumour suppressor in gastrointestinal cancers.^12^, ^13^, ^14^; RNF183 modulates inflammatory responses and ER stress signalling^15^, ^16^, ^17^, ^18^; and RNF24 is involved in membrane trafficking.^19^ Despite its inclusion in this family, the function of RNF39 has remained largely unexplored in cancer.

Notably, RNF39 has been implicated in the regulation of antiviral immunity and autoimmune pathogenesis. Genome‐wide association studies have linked RNF39 variants to HIV‐1 disease progression, multiple sclerosis, systemic lupus erythematosus and allergic rhinitis.^20^, ^21^, ^22^, ^23^ It also acts as a feedback suppressor of RIG‐I‐mediated innate immune signalling.^24^ Although prior work highlights the immunomodulatory capacity of RNF39, whether it governs proteostasis or ER stress in cancer − especially in epithelial tumours such as COAD − remains unclear.

RINT1 (RAD50‐interacting protein 1) acts as a multifunctional hub that links ER homeostatic pathways to oncogenic signalling, with defined roles in ER–Golgi trafficking and telomere maintenance, as well as contributions to the DNA‐damage response and cell‐cycle control.^25^, ^26^, ^27^, ^28^ RINT1 has been implicated as a context‐dependent tumour suppressor, yet somatic mutations and dysregulated expression have been associated with colorectal tumorigenesis.^25^, ^29^ Importantly, whether RINT1 stability is regulated post‐translationally and how such regulation affects ER stress responses in COAD remains unknown.

Although RNF39 has primarily been characterized in the context of immune regulation and viral response,^20^, ^24^, ^30^ its potential involvement in cancer biology may derive from its broader role in ubiquitin‐dependent proteostasis. The ER serves as a convergence point between immune signalling and tumour cell stress adaptation.^9^, ^31^ Colorectal cancer cells, which are frequently exposed to hypoxia, nutrient limitation and inflammatory cytokines within the tumour microenvironment,^1^, ^32^ may co‐opt immune‐related E3 ligases such as RNF39 to fine‐tune ER homeostasis and evade stress‐induced apoptosis. Thus, the oncogenic repurposing of RNF39 in COAD likely reflects a context‐dependent exploitation of an evolutionarily conserved immune–stress regulatory mechanism.

In this study, we characterize RNF39, a RING‐type E3 ligase previously linked to immune regulation, as a novel oncogenic regulator in colorectal cancer. We demonstrate that RNF39 promotes disease progression through ubiquitin‐mediated degradation of RINT1. We show that RNF39 is transcriptionally activated by MEF2D and enhances tumour cell proliferation and invasion by suppressing RINT1 accumulation, thereby attenuating UPR activation and preventing ER stress‐induced apoptosis. Mechanistically, we define a MEF2D–RNF39–RINT1 axis that integrates transcriptional control, proteostasis regulation and cell survival pathways in the context of ER stress. We provide mechanistic insight into the subversion of ER stress by colorectal cancer cells and identify RNF39 as a tractable target for therapy.

## 2 MATERIALS AND METHODS

### 2.1 Data sources and differential expression analysis

To investigate RNF39 expression and its relevance in COAD, we performed transcriptomic analyses using publicly available datasets from The Cancer Genome Atlas (TCGA) and Genotype‐Tissue Expression (GTEx) databases. COAD RNA‐sequencing data, including both tumour and normal tissue samples, were accessed through the GEPIA2 platform (http://gepia2.cancer‐pku.cn) for initial expression comparison and clinical correlation analyses. To ensure cross‐cohort validation, three independent Gene Expression Omnibus (GEO) datasets − GSE23878, GSE32323 and GSE110225 − were downloaded from the NCBI GEO database. Expression matrices were normalized and log2‐transformed using limma and edgeR in R (v4.3.1). Differential expression between tumour and matched adjacent tissue was assessed, designating genes as differentially expressed (DE) when |log_2_FC| > 1 and Benjamini–Hochberg FDR < .05. Visualization employed volcano plots and heatmaps generated with ggplot2, pheatmap and ComplexHeatmap. To profile RNF‐family dysregulation in COAD, a curated catalogue of RNF protein genes was overlapped with the upregulated DE sets from each dataset; candidates present across all datasets were defined as common hits and summarized using VennDiagram. Clinical parameters, including tumour stage, lymph node metastasis status and race, were assessed for association with RNF39 expression using the UALCAN database (http://ualcan.path.uab.edu). Overall survival (OS) and recurrence‐free survival (RFS) were evaluated using the Kaplan–Meier Plotter (http://kmplot.com); patients were stratified by RNF39 expression, and significance was assessed with the log‐rank test.

### 2.2 Cell culture and reagents

HCT116 and SW480 human colorectal cancer cell lines were obtained from the American Type Culture Collection and verified by short tandem repeat profiling. HCT116 cells were maintained in McCoy's 5A (KGL1701‐500, KeyGEN BioTECH), whereas SW480 cells were cultured in IMDM (KGL1101‐500, KeyGEN BioTECH); both media contained 10% FBS (A5669701, Gibco) and 1% penicillin–streptomycin (15140122, Thermo Fisher Scientific). Cells were cultured at 37°C in a humidified 5% CO_2_ atmosphere using a BB150 CO_2_ incubator (Thermo Fisher Scientific). ER stress was induced by treating cultures with thapsigargin (Sigma‐Aldrich, T9033) at 1 µM for the experiment‐specific time intervals.

### 2.3 ShRNA‐mediated knockdown

Lentiviral particles for knockdown were generated by co‐transfecting HEK293T cells with shRNA plasmids targeting RNF39 or RINT1, the packaging plasmid psPAX2 and the envelope plasmid pMD2.G (Addgene #12259), using Lipofectamine 3000 (Invitrogen, L3000015). At 48 h after transfection, culture supernatants were collected, clarified through a.45‐µm syringe filter and used immediately for infection. COAD cells seeded in 6‐well plates were transduced in the presence of polybrene (Sigma‐Aldrich, TR‐1003) to facilitate viral entry. After 48 h, cells were selected with puromycin (2 µg/mL; InvivoGen, ANT‐PR‐1) for 72 h to establish stable knockdown populations. Lentiviral vectors were purchased from Merck (Sigma).

### 2.4 CRISPR‐Cas9‐mediated knockout

CRISPR–Cas9 editing was used to establish RNF39^−^/^−^ colorectal carcinoma cell lines. Guide RNAs targeting exon 1 of human RNF39 were selected using the Zhang Lab online CRISPR design portal (https://crispr.mit.edu). Complementary oligonucleotides encoding the sgRNA were phosphorylated, annealed and ligated into the BbsI sites of pSpCas9(BB)‐2A‐Puro (PX459) v2.0 (Addgene #62988). HCT116 and SW480 cells were transfected with the PX459–sgRNF39 constructs using Lipofectamine 3000 (Invitrogen, L3000015). At 48 h post‐transfection, cells underwent puromycin selection for 72 h to enrich edited populations. Surviving cells were then plated by limiting dilution into 96‐well plates to derive single‐cell clones. Expanded clones underwent PCR genotyping, qRT‐PCR and immunoblotting, which verified RNF39 knockout and biallelic loss of expression.

### 2.5 Quantitative PCR

Following RNA extraction from cultured cells using TRIzol reagent (Invitrogen, 15596018), 1 µg of quantified RNA (NanoDrop 2000, Thermo Fisher Scientific) was reverse‐transcribed into cDNA with the High‐Capacity cDNA Reverse Transcription Kit (Applied Biosystems, 4368814). qPCR was performed in 20 µL reactions on an ABI 7500 Fast system (Applied Biosystems) using PowerUp SYBR Green Master Mix (Thermo Fisher Scientific, A25742), 250 nM primers and 2 µL cDNA. The protocol consisted of initial denaturation (95°C, 2 min) and 40 cycles of 95°C for 15 s and 60°C for 60 s. Relative expression was computed by 2^−ΔΔCt with normalization to GAPDH or ACTB.

### 2.6 Western blotting

Cell lysates prepared in RIPA buffer (Thermo Fisher Scientific, 89900) with protease inhibitors (Roche, 11873580001) on ice were centrifuged (14 000 × *g*, 15 min, 4°C) to collect supernatants for protein quantification by BCA assay (Thermo Fisher Scientific, 23227). Proteins (20–40 µg) were separated by SDS–PAGE (10–12%), transferred to PVDF membranes (Millipore, IPVH00010) and blocked with 5% non‐fat milk. After incubation with primary antibodies (4°C, overnight) and HRP‐conjugated secondary antibodies (room temperature, 45 min), signals were detected with Tanon ECL reagent using a Tanon 5200 imaging system.

### 2.7 Immunoprecipitation and co‐immunoprecipitation

Cells were lysed in ice‐cold RIPA buffer (4°C, 60 min) and then sonicated on ice for 10 min. The lysate was then clarified by a 3‑min spin at 15 000 rpm, and the supernatant was recovered. For immunoprecipitation (IP), clarified supernatants were incubated with anti‐RNF39 or anti‐RINT1 antibodies (5 µg/mL) at 4°C overnight. Immune complexes were captured with Protein A+G magnetic beads for 1 h at room temperature, washed in PBS and released by boiling in SDS loading buffer (95°C, 10 min). RNF39 and RINT1 were subsequently detected by western blotting. For co‐immunoprecipitation (co‐IP), cell lysates were precleared with mouse IgG–coated beads for 3 h at 4°C, followed by overnight incubation with the designated primary antibodies at 4°C. The bead‐bound immunocomplexes were then thoroughly washed, eluted by boiling in SDS sample buffer at 95°C for 10 min, and subsequently analysed by immunoblotting for the proteins of interest.

### 2.8 Ubiquitination assays

To stabilize ubiquitinated proteins, cells were pretreated with the proteasome inhibitor MG132 before harvest. For TUBE2 pulldown, sh‐NC– or sh‐RNF39–infected cells were lysed in RIPA buffer with protease and phosphatase inhibitors, incubated with TUBE2 (Tandem Ubiquitin Binding Entity 2) agarose, and the enriched fractions were immunoblotted to detect ubiquitinated RINT1, with RNF39 and β‐actin in the input serving as controls. Ubiquitination of RINT1 in HEK293T cells was examined by co‐transfecting HA‐RINT1, Flag‐RNF39 (or vector) and His‐tagged K48‐only ubiquitin (His‐K48‐Ub), followed by anti‐HA IP and immunoblotting with anti‐His antibodies. Expression of HA‐RINT1 and Flag‐RNF39 was verified in input samples. For in vitro ubiquitination, lysates from HEK293T cells expressing HA‐RINT1 and/or Flag‐RNF39 were incubated in ubiquitination buffer containing recombinant E1, E2, ATP and wild‐type (WT) ubiquitin. Reactions lacking RNF39 and/or E1/E2 served as negative controls. After anti‐HA IP, RINT1–ubiquitin conjugates were detected by anti‐HA immunoblotting.

### 2.9 EdU assay

Cells were pulsed with 5–10 µM EdU in complete medium for 1–2 h at 37°C under 5% CO_2_ prior to fixation with 4% paraformaldehyde for 15–30 min. Residual aldehydes were neutralized with 2 mg/mL glycine for 5 min at RT, washed in PBS containing 3% BSA, and cells were permeabilized with.5% Triton X‐100 for 20 min at RT before EdU detection (YEASEN, 40276ES60). Fluorescent signals were captured by fluorescence microscopy to evaluate proliferation.

### 2.10 Colony formation, invasion and wound‐healing assays

Clonogenic assays were performed by seeding cells in 12‐well plates for 14 days, followed by fixation (4% paraformaldehyde, 15–30 min), crystal‐violet staining (20 min), air‐drying and ImageJ‐based quantification (colonies/area). For invasion, Matrigel‐coated Transwell inserts (NEST, 725331) were used; cells were loaded into the upper chamber in serum‐free medium, with 10% FBS medium in the lower chamber as the attractant. After 24–48 h, cells on the lower surface of the membrane were fixed, stained, imaged on an inverted microscope (Nikon) and analysed with ImageJ. For wound‐healing (migration), confluent monolayers in 12‐well plates were wounded with a sterile tip; debris was removed by PBS washes, cultures were maintained in serum‐free medium, and wound closure was recorded at indicated times (e.g. 0 and 24–48 h) and quantified in ImageJ.

### 2.11 In vivo tumour growth assays

Female BALB/c nude mice (4–6 weeks) were obtained from the National Rodent Laboratory Animal Resources Center and maintained under specific pathogen‐free conditions in accredited facilities (22 ± 1°C; 50–60% relative humidity; 12‐h light/dark cycle) with ad libitum access to autoclaved chow and water. For xenografts, cells expressing shRNF39, shRINT1 or combined shRNF39/shRINT1 were resuspended in sterile PBS and injected subcutaneously into the right flank (*n* = 5 per group). Tumour dimensions were recorded every 3 days using a digital calliper, and volumes were calculated as (length × width^2^)/2. After 4 weeks, mice were euthanized by CO_2_ inhalation; tumours were excised, photographed and weighed. Specimens were fixed in 10% neutral‐buffered formalin for histology or snap‐frozen in liquid nitrogen for subsequent protein and RNA analyses.

### 2.12 Chromatin immunoprecipitation assays

Chromatin immunoprecipitation (ChIP) was conducted with a commercial kit (#17‐295, MilliporeSigma). Cells were crosslinked with formaldehyde to preserve protein–DNA complexes and then sonicated to shear chromatin. The fragmented chromatin underwent IP using antibodies specific for MEF2D or an irrelevant IgG control. DNA was extracted from the immunoprecipitated complexes after incubation and precipitation. PCR was performed on purified DNA using RNF39‐specific primers, with GAPDH as the control. Amplicons were resolved by agarose gel electrophoresis to assess enrichment of the target sequences.

### 2.13 Luciferase activity

To investigate MEF2D‐dependent transcriptional control of RNF39, HEK293T cells were transfected with pGL4.15 vectors harbouring either WT or mutant (MUT) RNF39 promoters together with a Renilla luciferase control (pGL4.15‐Renilla), with or without co‐transfection of MEF2D. The vectors contained defined promoter elements, including the SV40 promoter (SV40p), and an MCS permitting insertion of variable promoters. Following transfection, luciferase activity in cell lysates was determined using the Dual‐Luciferase Reporter Assay System (Promega, E1910). Data are presented as the ratio of firefly to Renilla luminescence to control for transfection efficacy and cell viability.

### 2.14 Immunohistochemistry

COAD specimens were collected intraoperatively. Serial 4‐µm sections were then prepared from 10% formalin‐fixed, paraffin‐embedded tissue using a rotary microtome. Immunohistochemistry was carried out on a LEICA BOND‐III automated stainer. Whole‐slide images were captured with a digital pathology scanner (NanoZoomer S210). Protein expression was evaluated using a semi‐quantitative H‐score (0–300), calculated as H‐score = Σ (staining intensity × percentage of positive cells); intensity grades were 0 = negative, 1 = weak, 2 = moderate and 3 = strong. For quality control, two pathologists independently scored the slides in a double‐blind manner, and discrepant cases were reviewed by a third party and confirmed through a consensus meeting to determine the final results. The use of human tissue samples in this study was approved by the Ethics Committee of Ruijin Hospital, Shanghai Jiao Tong University School of Medicine (Approval No. KY2025022).

### 2.15 Statistical analysis

All experiments included at least three independent replicates, and data are shown as the mean ± SD. Analyses were conducted in GraphPad Prism 10.1.2. We used two‐tailed Student's *t*‐tests for two‐group comparisons and one‐way ANOVA with Tukey's post‐hoc test for multi‐group comparisons. Factorial designs were analysed by two‐way ANOVA with Tukey correction. A statistical significance threshold of *p* < .05 was defined, and the corresponding symbols are represented as *p < .05, **p < .01, ***p < .001 and ****p < .0001.

Primer sequences and the antibody list are provided in Table S1.

## 3 RESULTS

### 3.1 Integrated transcriptomic analysis identifies RNF39 as a consistently upregulated E3 ligase in colorectal cancer

**FIGURE 1:**
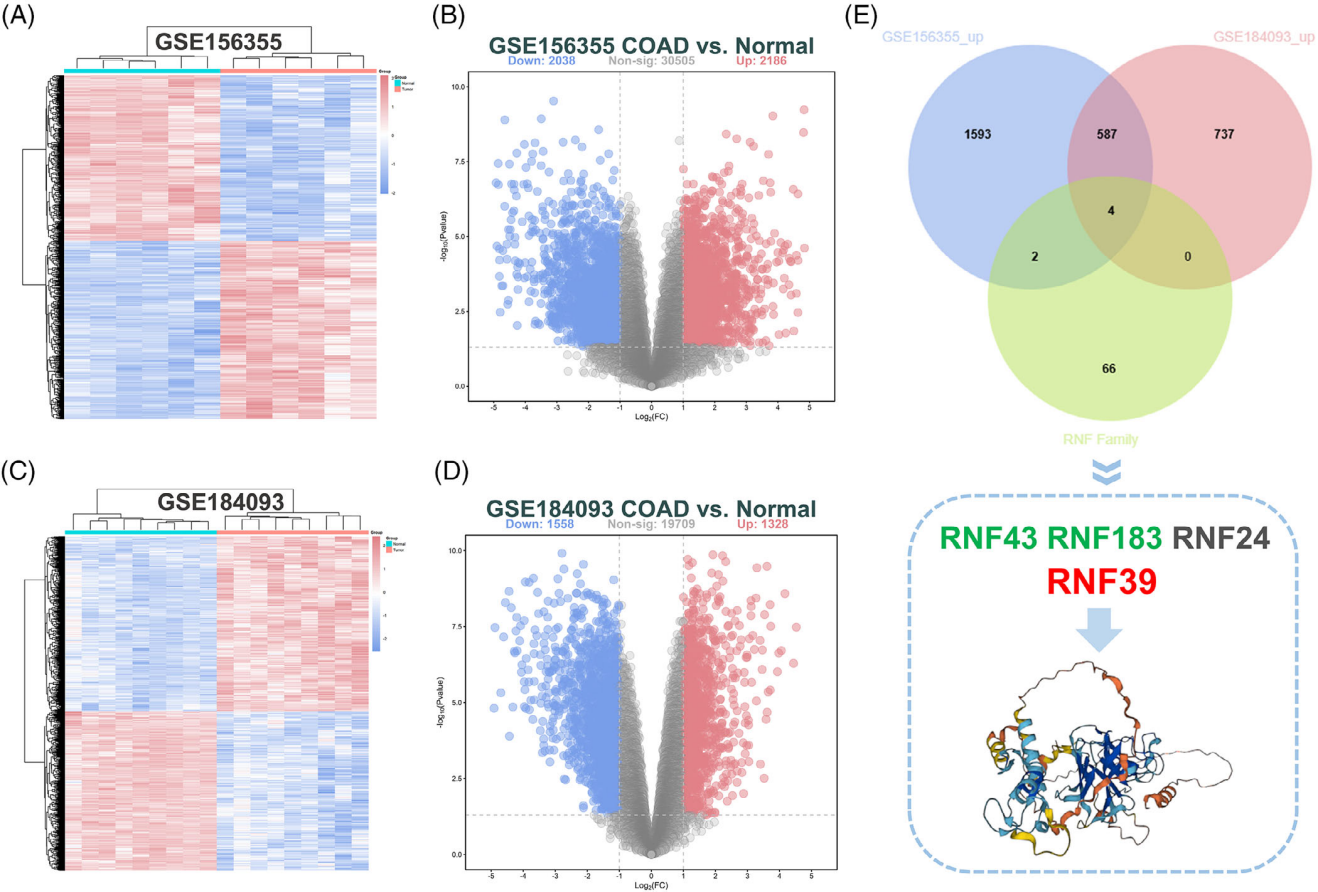
Transcriptomic profiling identifies RNF39 as a novel upregulated RNF family gene in COAD. (A) Heatmap displaying differentially expressed genes (DEGs) between colorectal adenocarcinoma (COAD) and adjacent normal tissues in the GSE156355 dataset. (B) Volcano plot showing the differential expression between COAD and normal tissues in the GSE156355 dataset, with 2038 downregulated and 30 505 non‐significant genes, and 2186 upregulated genes. Differential expression was determined using a cutoff of adjusted p‐value < .05 and |log2 fold change| > 1.0. (C) Heatmap illustrating DEGs between tumour and normal tissues in the GSE184093 dataset. (D) Volcano plot depicting the differential expression between COAD and normal tissues in the GSE184093 dataset, with 1558 downregulated and 19 709 non‐significant genes, and 1328 upregulated genes, using the same statistical criteria. (E) Venn diagram showing the overlap of upregulated genes from GSE156355 and GSE184093 with a reference list of RNF (RING finger protein) family genes. The intersection includes RNF43, RNF183, RNF24 and RNF39.

To elucidate the transcriptional dysregulation underlying COAD, we performed differential gene expression analysis using two independent GEO datasets, GSE156355 and GSE184093. Hierarchical clustering and heatmap visualization revealed a clear separation between tumour and adjacent normal tissues, reflecting distinct transcriptional landscapes (Figure 1A,C). In the dataset GSE156355, differential expression analysis identified 2186 upregulated and 2038 downregulated genes in tumours relative to normal tissues (|log2FC| > 1.0, *p* < .05; Figure 1B). Similar patterns were observed in GSE184093, where 1328 genes were upregulated, and 1558 were downregulated (Figure 1D), underscoring the reproducibility of transcriptomic alterations across cohorts. Given the emerging roles of E3 ubiquitin ligases in cancer biology, we focused on the RNF protein family. Venn diagram analysis identified four RNF genes − RNF43, RNF183, RNF24 and RNF39 − that were consistently upregulated in both datasets (Figure 1E). While RNF43 and RNF183 have been previously implicated in colorectal cancer progression,^12^, ^13^, ^14^, ^18^ the consistent and significant elevation of RNF39, a comparatively understudied member of this protein family, suggests it may function as a novel and distinct regulator in COAD pathogenesis.

### 3.2 RNF39 expression is elevated in COAD across multiple datasets and clinical subgroups

**FIGURE 2:**
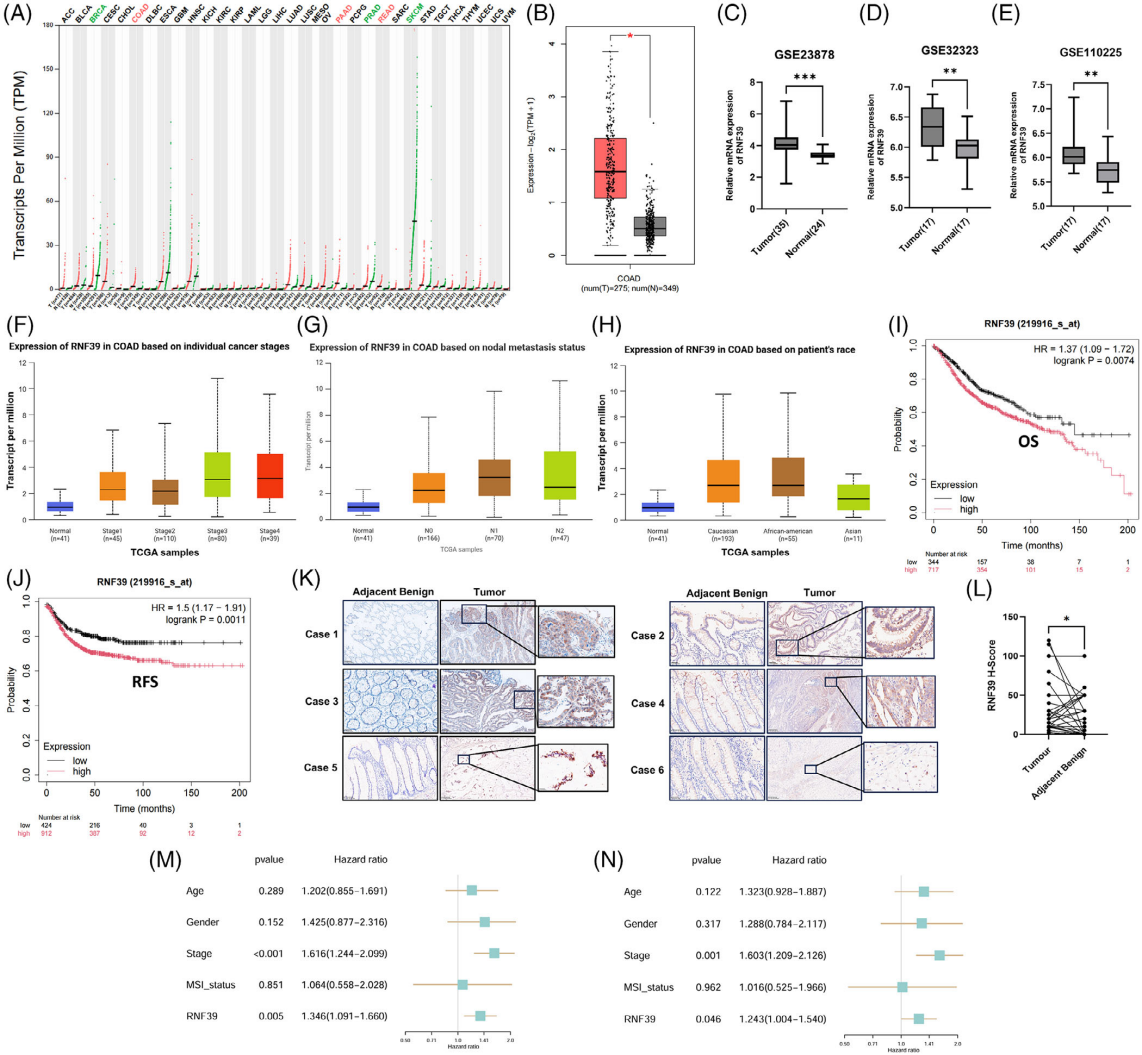
RNF39 expression patterns in colorectal adenocarcinoma across public datasets and clinical subgroups. (A) Pan‐cancer analysis of RNF39 transcript levels across 33 tumour types using TCGA and GTEx datasets. Data are represented as transcripts per million (TPM). (B) Box plot comparing RNF39 mRNA expression between colon adenocarcinoma (COAD, *n* = 275) and normal colon tissues (*n* = 349) based on TCGA and GTEx integration using the GEPIA platform. (C–E) RNF39 expression in tumour and normal colon tissues across three independent GEO datasets: GSE23878 (C), GSE32323 (D) and GSE110225 (E). (F) RNF39 transcript levels in COAD samples from TCGA stratified by tumour stage. (G) RNF39 expression levels categorized by lymph node metastasis status (N0–N2) in COAD patients. (H) RNF39 transcript levels in COAD samples stratified by patient race (Caucasian, African American, Asian). (I, J) Kaplan–Meier survival curves evaluating overall survival (OS, I) and recurrence‐free survival (RFS, J) in COAD patients, stratified by RNF39 expression levels. Data were obtained using the Kaplan–Meier Plotter database. (K) Immunohistochemical (IHC) staining for RNF39 in six representative colorectal adenocarcinoma cases. (L) *T*‐test analysis of RNF39 expression H‐scores between adjacent normal tissues and tumour tissues. (M) Univariate Cox proportional hazards analysis of overall survival in the TCGA‐COAD cohort for age, gender, tumour stage, MSI status and RNF39 expression. (N) Multivariable Cox proportional hazards model including age, gender, tumour stage, MSI status and RNF39 expression, demonstrating that high RNF39 remains an independent unfavourable prognostic factor in COAD.

To further assess RNF39 expression patterns in human malignancies, we performed a pan‐cancer transcriptomic analysis based on TCGA and GTEx datasets. Among 33 tumour types analysed, elevated RNF39 transcript levels were observed in COAD compared to normal tissues (Figure 2A). Quantitative analysis using the GEPIA platform confirmed significantly higher RNF39 expression in 275 COAD samples relative to 349 normal colon samples (Figure 2B). This expression pattern was consistently observed in three independent GEO datasets. RNF39 mRNA expression was significantly upregulated in tumour tissues across multiple datasets: GSE23878 (tumour *n* = 35, normal *n* = 24), GSE32323 (tumour *n* = 17, normal *n* = 17) and GSE110225 (tumour *n* = 17, normal *n* = 17) (Figure 2C–E). Stratified analyses based on TCGA clinical annotations were conducted to examine the distribution of RNF39 expression across different clinical parameters. RNF39 transcript levels were elevated in tumour samples at stages I through IV compared to normal tissues (Figure 2F). A similar trend was observed when samples were grouped by lymph node metastasis status, with increased RNF39 expression across N0, N1 and N2 categories (Figure 2G). When grouped by patient race, RNF39 expression appeared higher in COAD tissues from Caucasian and African American patients compared to those from Asian patients (Figure 2H). Quantification of staining scores in matched samples further supported increased RNF39 protein expression in tumour tissues. Kaplan–Meier survival analysis using publicly available datasets revealed differences in OS and RFS between patients with high and low RNF39 expression (Figure 2I,J). These results suggest that RNF39 expression varies across disease states and clinical subgroups in COAD. We validated RNF39 protein expression in COAD by immunohistochemistry using a clinical cohort of 58 cases with diverse histological types. Overall, RNF39 protein expression was significantly elevated in tumour tissues relative to adjacent normal tissues, as determined by immunohistochemical staining and H‐score. Representative cases of RNF39 overexpression across different histological subtypes, including tubular adenocarcinoma (Cases 1–4), mucinous adenocarcinoma (Case 5) and signet‐ring cell carcinoma (Case 6) (Figure 2K). Further, *t*‐test analysis of RNF39 expression H‐scores between adjacent normal tissues and tumour tissues showed statistical significance (Figure 2L). To assess whether RNF39 is an independent prognostic factor in COAD, we performed Cox proportional hazards analysis in the TCGA‐COAD cohort using age, gender, tumour stage, MSI status and RNF39 expression as covariates (Figure 2M,N). In the multivariable model, advanced tumour stage and high RNF39 expression remained significantly associated with poorer overall survival, indicating that RNF39 acts as an independent unfavourable prognostic factor in COAD.

### 3.3 MEF2D is associated with transcriptional regulation of RNF39 in COAD

**FIGURE 3:**
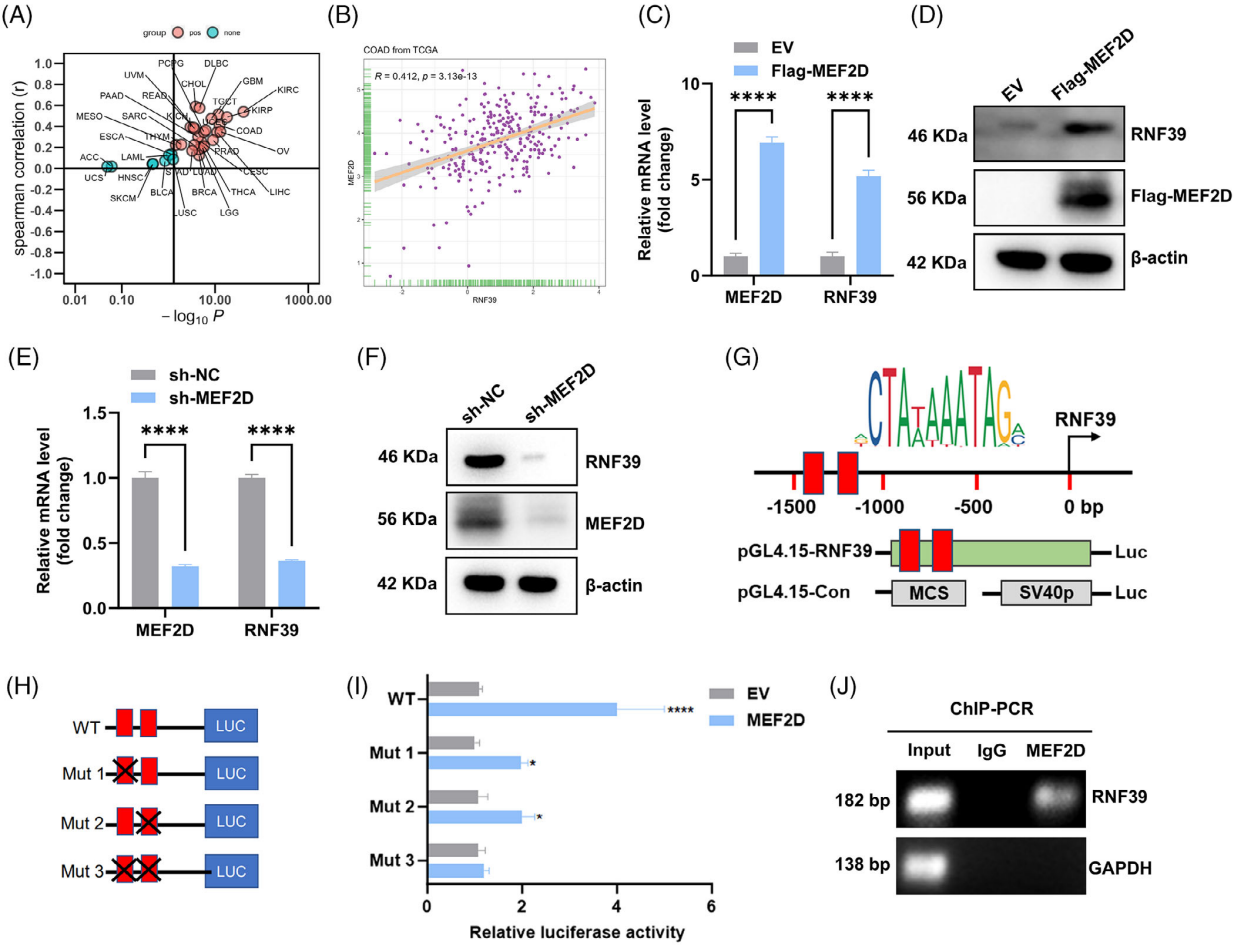
MEF2D regulates RNF39 expression in COAD. (A) Spearman correlation analysis of RNF39 expression with the top 50 most correlated genes in the TCGA COAD dataset, highlighting MEF2D as a significant positive correlate. (B) Correlation plot showing the relationship between MEF2D and RNF39 mRNA expression in COAD samples from TCGA, with Spearman's correlation coefficient (*r*) and statistical significance indicated. (C, D) qRT‐PCR (C) and western blot (D) analysis of RNF39 and MEF2D expression in COAD cells transfected with Flag‐tagged MEF2D (Flag‐MEF2D) or empty vector (EV). (E, F) qRT‐PCR (E) and western blot (F) analysis of RNF39 and MEF2D expression in COAD cells transfected with shRNA targeting MEF2D (sh‐MEF2D) or non‐targeting control (sh‐NC). (G) Diagram of the RNF39 promoter region showing the location of potential MEF2D response elements (−1400 to −1000 bp) identified by the JASPAR database. (H) Schematic of the luciferase reporter constructs used to assess RNF39 promoter activity, including wild‐type (WT) and mutant (Mut 1, Mut 2, Mut 3) response elements. (I) Luciferase reporter assay results showing relative luciferase activity in COAD cells transfected with WT or mutant RNF39 promoter constructs, with and without MEF2D overexpression. (J) Chromatin immunoprecipitation (ChIP)‐PCR analysis showing MEF2D binding to the RNF39 promoter region (−1400 to −1000 bp) in COAD cells. Input and IgG are shown as controls.

To identify potential transcriptional regulators of RNF39 in COAD, a correlation analysis was performed using the TCGA‐COAD transcriptome dataset. MEF2D was the transcription factor most positively correlated with RNF39 expression among the top 50 associated genes (Figure 3A). The significant positive correlation between MEF2D and RNF39 expression was validated in the COAD cohort (Spearman *r* = .412, *p* = 3.13 × 10^−13^; Figure 3B). To experimentally investigate this regulatory relationship, we transfected COAD cells with a plasmid encoding Flag‐tagged MEF2D. Overexpression of MEF2D led to a significant increase in RNF39 mRNA and protein levels compared to empty vector controls (Figure 3C,D). Conversely, MEF2D knockdown mediated by shRNA, both RNF39 transcript and protein levels were significantly attenuated (Figure 3E,F). To explore potential MEF2D response elements within the RNF39 promoter, the upstream regulatory sequence (−1500 to 0 bp) was analysed using the JASPAR database. Two MEF2D binding motifs were identified within the −1400 to −1000 bp region (Figure 3G). A 1.5 kb fragment of the RNF39 promoter was cloned into a luciferase reporter plasmid, and luciferase assays demonstrated increased promoter activity upon MEF2D overexpression compared to vector controls. Mutational analysis of individual or combined MEF2D binding sites showed progressive attenuation of luciferase activity, indicating these two elements both contribute to transcriptional activation (Figure 3H,I). ChIP assays further confirmed that MEF2D is enriched at the RNF39 promoter region in COAD cells, as shown by PCR amplification following MEF2D IP (Figure 3J).

### 3.4 RNF39 promotes the proliferation and invasion of colorectal cancer cells in vitro

**FIGURE 4:**
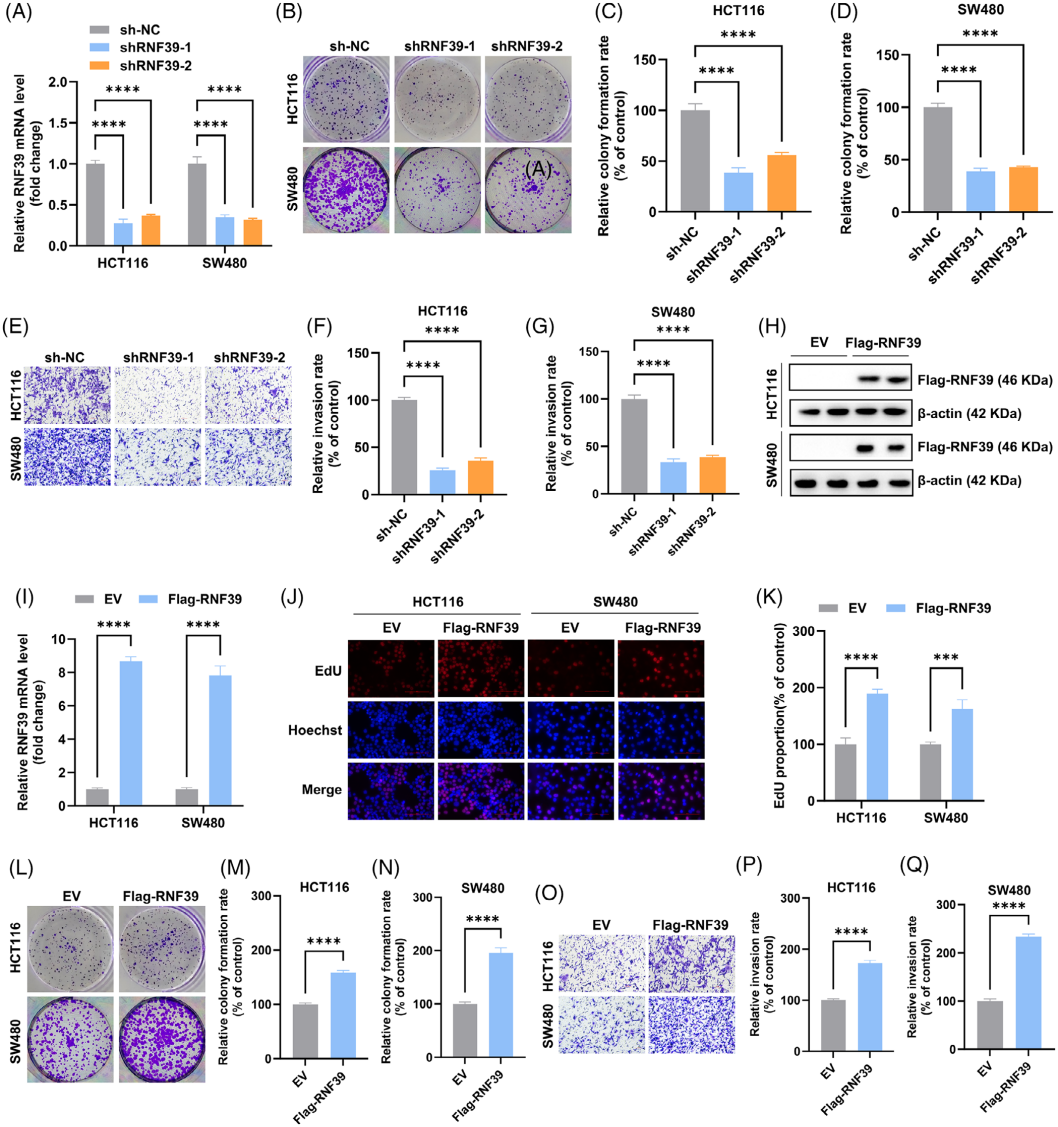
RNF39 regulates proliferation and invasion in COAD cells. (A) RNF39 mRNA expression in HCT116 and SW480 cells transfected with two independent shRNAs targeting RNF39 (shRNF39‐1 and shRNF39‐2) or a negative control (sh‐NC), assessed by qRT‐PCR. (B) Representative images of colony formation assays in RNF39‐knockdown and control cells. (C, D) Quantification of colony numbers in HCT116 (C) and SW480 (D) cells following RNF39 knockdown. (E) Representative images of transwell invasion assays in RNF39‐silenced and control cells. (F, G) Quantification of invaded cells in HCT116 (F) and SW480 (G) after RNF39 knockdown. (H, I) Western blot (H) and qRT‐PCR (I) validation of RNF39 overexpression in HCT116 and SW480 cells transfected with Flag‐RNF39 or empty vector (EV). (J) Representative images of EdU staining in control and RNF39‐overexpressing cells; Hoechst was used to stain nuclei. (K) Quantification of EdU‐positive cells in HCT116 and SW480 cells. (L) Representative images of colony formation assays following RNF39 overexpression. (M, N) Quantification of colony numbers in HCT116 (M) and SW480 (N) cells with or without RNF39 overexpression. (O) Representative images of transwell invasion assays in RNF39‐overexpressing and control cells. (P, Q) Quantification of invaded cells in HCT116 (P) and SW480 (Q) with or without RNF39 overexpression.

The functional role of RNF39 in COAD was investigated through loss‐of‐function and gain‐of‐function experiments in HCT116 and SW480 cell lines. We employed two independent shRNAs (shRNF39‐1 and shRNF39‐2) targeting RNF39 to achieve efficient gene knockdown. Quantitative RT‐PCR confirmed efficient knockdown of RNF39 mRNA expression in both cell lines relative to the non‐targeting control (Figure 4A). In colony formation assays, RNF39 depletion significantly suppressed clonogenic potential, as reflected by reduced colony number and size in both HCT116 and SW480 cells (Figure 4B–D). Transwell invasion assays further revealed that silencing RNF39 resulted in a marked decrease in invasive capacity, suggesting a role in promoting extracellular matrix traversal (Figure 4E–G). To complement the knockdown studies, we established RNF39‐overexpressing cell lines via transfection with a Flag‐tagged RNF39 construct. RNF39 knockdown efficiency was verified at both the protein (immunoblotting) and mRNA (qRT‐PCR) levels (Figure 4H,I). Functional assays demonstrated that RNF39 overexpression enhanced proliferative activity in both cell lines. EdU incorporation assays showed a significant increase in S‐phase cells upon RNF39 overexpression, as indicated by elevated EdU‐positive nuclear staining compared to empty vector controls (Figure 4J,K). In parallel, colony formation assays revealed that RNF39‐overexpressing cells generated substantially more colonies than controls (Figure 4L–N), consistent with increased long‐term proliferative capacity. Invasion assays were also conducted to assess whether RNF39 contributes to the invasive phenotype. Compared to control cells, RNF39‐overexpressing HCT116 and SW480 cells exhibited enhanced transwell migration through Matrigel‐coated chambers (Figure 4O–Q), supporting a role for RNF39 in facilitating cellular invasiveness. Collectively, these in vitro data indicate that modulation of RNF39 expression significantly influences key tumour‐associated phenotypes in colorectal cancer cells, including proliferation, clonogenicity and invasive behaviour.

### 3.5 RNF39's E3 ligase activity is required for its tumour‐promoting functions in COAD

**FIGURE 5:**
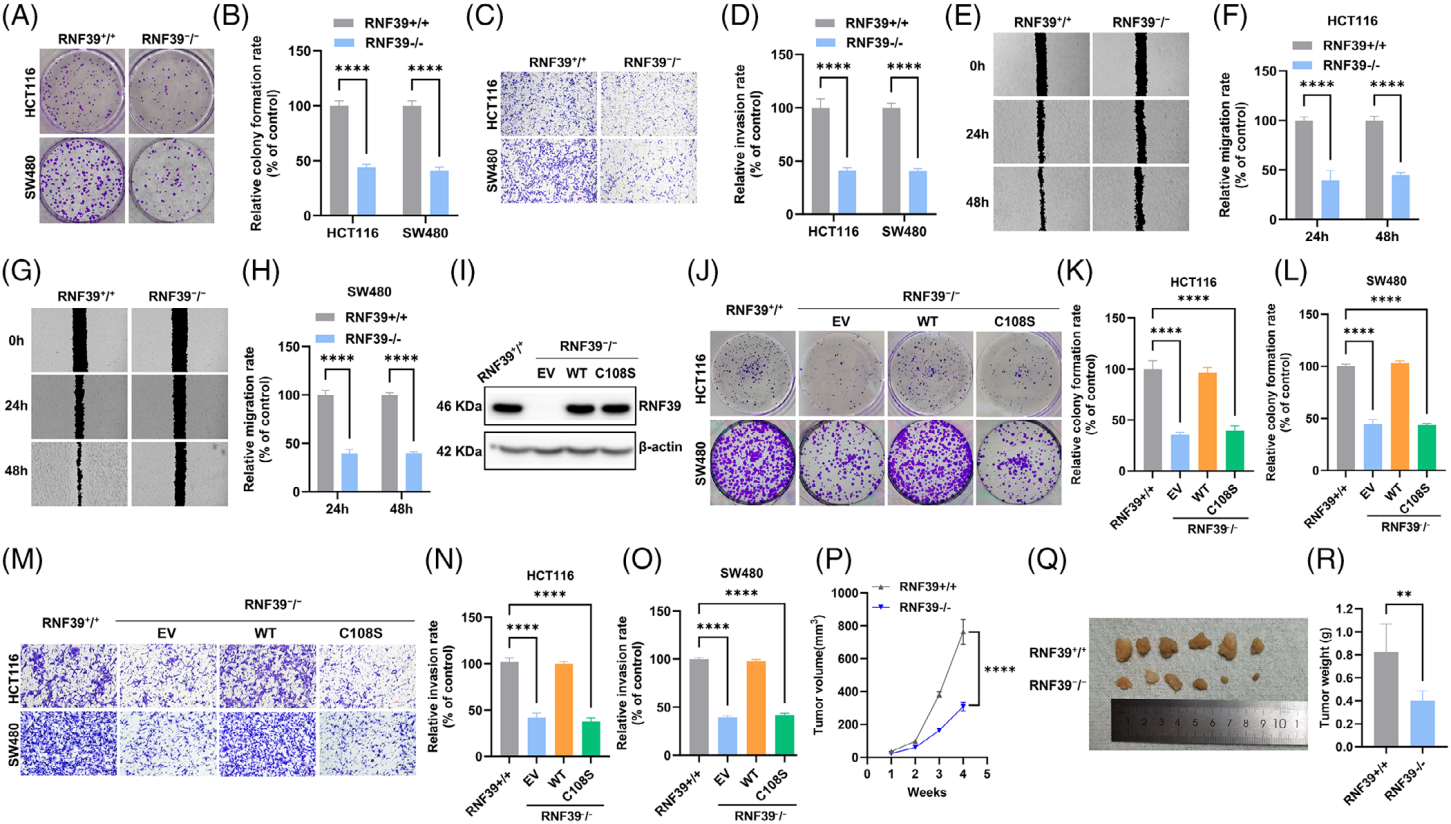
RNF39 E3 ligase activity is required for COAD cell oncogenicity and tumour growth in vivo. (A) Representative images of colonies formed by RNF39⁺/⁺ and RNF39^−^/^−^ HCT116 and SW480 cells in a colony formation assay. (B) Quantification of the number of colonies in A. (C) Representative images of invaded RNF39⁺/⁺ and RNF39^−^/^−^ HCT116 and SW480 cells in a Matrigel‐coated transwell assay. (D) Quantification of invaded cells in C. (E) Representative images of wound closure in RNF39⁺/⁺ and RNF39^−^/^−^ HCT116 cells at 0, 24 and 48 h in a wound healing assay. (F) Quantification of the relative migration rate in E. (G) Representative images of wound closure in RNF39⁺/⁺ and RNF39^−^/^−^ SW480 cells in a wound healing assay. (H) Quantification of the relative migration rate in G. (I) Immunoblot validation of RNF39 expression in wild‐type (RNF39⁺/⁺) and knockout (RNF39^−^/^−^) HCT116 and SW480 cells reconstituted with empty vector (EV), wild‐type RNF39 (WT) or catalytically inactive mutant RNF39^C108S. (J) Representative colony formation assay images from the indicated HCT116 and SW480 cell groups. (K, L) Quantification of relative colony numbers in HCT116 (K) and SW480 (L) RNF39^−^/^−^ cells reconstituted with EV, WT or C108S. (M) Representative images of transwell invasion assays in the indicated cell groups. (N, O) Quantification of invaded cells in HCT116 (N) and SW480 (O) cells from transwell assays. (P) Tumour growth curves showing volume measurements over time in nude mice subcutaneously injected with RNF39⁺/⁺ or RNF39^−^/^−^ HCT116 cells (*n* = 5 per group). (Q) Representative image of excised tumours from both groups at endpoint. (R) Quantification of tumour weights at harvest from RNF39⁺/⁺ and RNF39^−^/^−^ xenografts.

To examine whether the E3 ubiquitin ligase activity of RNF39 contributes to its tumour‐promoting effects, RNF39 knockout (RNF39^−^/^−^) HCT116 and SW480 cells were generated using CRISPR‐Cas9 genome editing. WT RNF39 or a catalytically inactive MUT (RNF39^C108S, carrying a cysteine‐to‐serine substitution in the RING domain) was reintroduced into RNF39^−^/^−^ cells via stable transfection, alongside an empty vector control. We found that RNF39^−^/^−^ cells exhibited a significant impairment in clonogenic growth (Figure 5A,B) and a reduced capacity for cell invasion (Figure 5C,D) compared to their RNF39⁺/⁺ counterparts. Furthermore, the migratory ability of RNF39^−^/^−^ cells was significantly attenuated, as evidenced by delayed wound closure in a scratch assay (Figure 5E–H), a phenotype that was similarly dependent on functional E3 ligase activity. Western blotting confirmed expression of WT and MUT RNF39 constructs (Figure 5I). Colony formation assays demonstrated a markedly attenuated clonogenic capacity in RNF39‐knockout cells relative to WT controls. Reintroduction of WT RNF39, but not the C108S MUT, rescued colony‐forming ability in RNF39^−^/^−^ cells (Figure 5J–L). Similarly, transwell invasion assays revealed that the invasive defect in RNF39^−^/^−^ cells was rescued by reintroducing WT RNF39, whereas the ligase‐inactive MUT was ineffective (Figure 5M–O). To evaluate these findings in vivo, xenograft assays were performed using RNF39⁺/⁺ and RNF39^−^/^−^ HCT116 cells implanted subcutaneously into immunodeficient nude mice. RNF39^−^/^−^ xenografts exhibited a markedly reduced tumour volume relative to WT controls (Figure 5P). Endpoint measurements confirmed significantly lower tumour weights in the RNF39^−^/^−^ cohort (Figure 5Q,R). The oncogenic effects of RNF39 on COAD cells − enhancing proliferation, invasion and tumour growth − are attributable to its E3 ubiquitin ligase function.

### 3.6 RINT1 is identified as a direct binding partner of RNF39 in COAD

**FIGURE 6:**
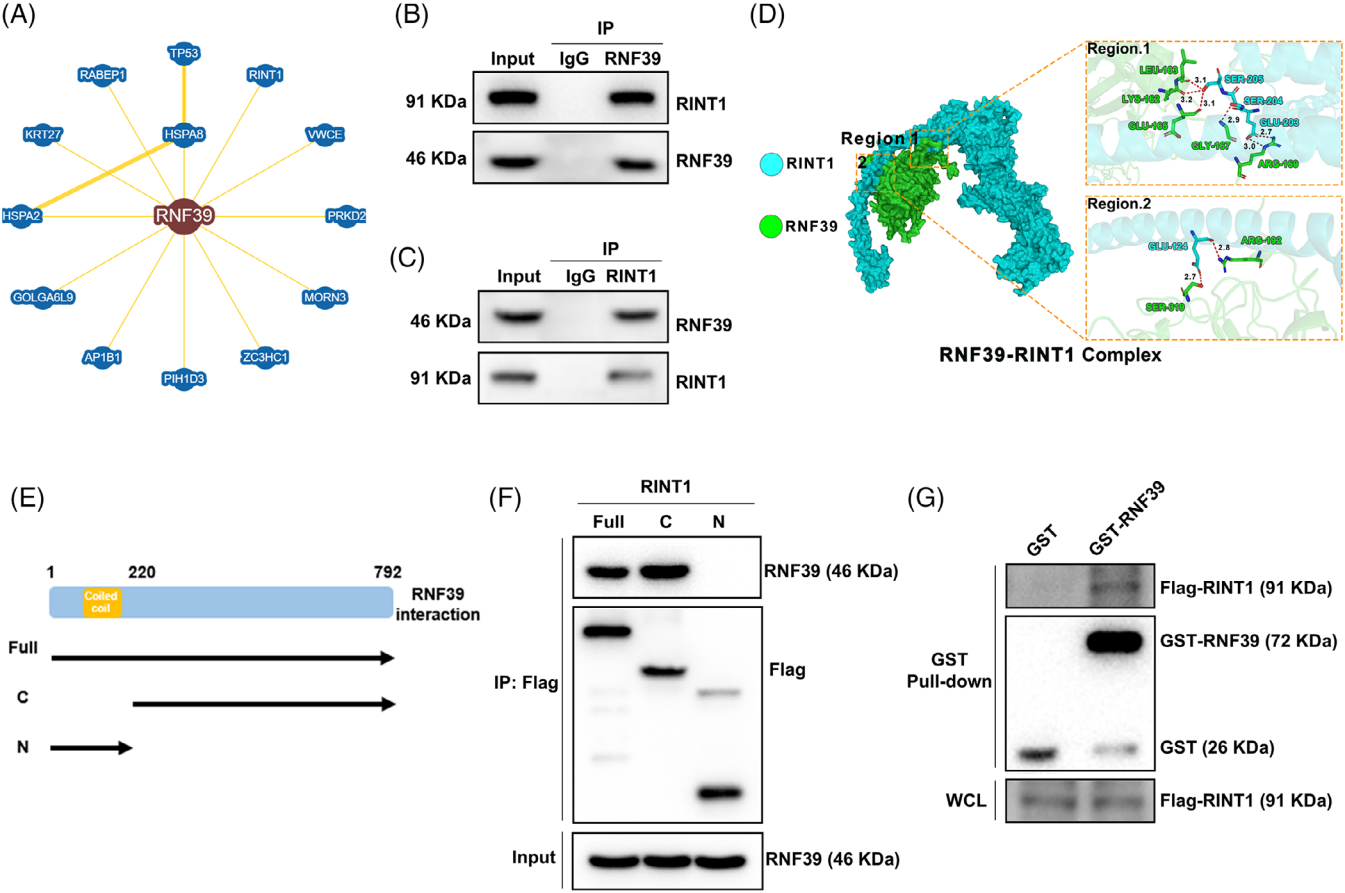
Identification and validation of RINT1 as a direct binding partner of RNF39 in colorectal adenocarcinoma. (A) Network diagram generated from the BioGRID database showing 13 candidate RNF39‐interacting proteins. RINT1 is highlighted for follow‐up validation. (B, C) Co‐immunoprecipitation (co‐IP) assays in COAD cells treated with MG132 (10 µM, 4 h). (B) RNF39 was immunoprecipitated, and RINT1 was detected by western blotting. (C) Reciprocal co‐IP showing RNF39 in complexes immunoprecipitated with anti‐RINT1 antibody. IgG was used as a negative control. (D) Predicted structure of the RNF39–RINT1 complex using AlphaFold2 and ClusPro docking. Zoom‐in views (bottom panels) show hydrogen bond networks at the predicted protein–protein interaction interface, rendered by PyMOL and LigPlot. (E) Schematic representation of the RINT1 deletion constructs (Full, C, N). (F) 293T cells were transfected with the indicated Flag‐tagged RINT1 deletion constructs for 36 h. Cell lysates were immunoprecipitated with anti‐Flag resin, followed by immunoblotting with the indicated antibodies. The Co‐IP results identify the C‐terminal domain of RINT1 as necessary and sufficient for its interaction with RNF39. (G) GST pulldown assays showing direct interaction between bacterially purified GST‐RNF39 and Flag‐RINT1 from whole‐cell lysates. GST alone was used as a control. WCL, whole‐cell lysate input.

To map the molecular interaction landscape of RNF39, we systematically queried BioGRID, which aggregates protein–protein interaction evidence from yeast two‐hybrid, co‐IP and affinity capture–mass spectrometry studies. This analysis identified 13 candidate interacting proteins, among which RINT1 was prioritized for further investigation based on its established role in ER homeostasis and vesicular trafficking, as well as its reported tumour‐suppressive function in other malignancies (Figure 6A). To validate the interaction between RNF39 and RINT1 at the endogenous level, co‐IP experiments were conducted in COAD cells pretreated with the proteasome inhibitor MG132 (10 µM, 4 h) to stabilize potential ubiquitin‐modified intermediates. IP with anti‐RNF39 antibody followed by immunoblotting revealed the presence of RINT1 in RNF39 complexes (Figure 6B). Reciprocal co‐IP using anti‐RINT1 antibody confirmed RNF39 detection in RINT1‐associated complexes, supporting a specific bidirectional interaction (Figure 6C). To obtain a putative structural hypothesis for this interaction, AlphaFold2‐predicted 3D models of human RNF39 and RINT1 were analysed by rigid‐body docking using ClusPro. These computational results are exploratory and serve to guide future biochemical validation. Analysis of the top‐ranking complex revealed multiple hydrogen bond‐mediated contacts at the predicted interface, visualized using LigPlot and PyMOL (Figure 6D). RINT1 features a characteristic N‐terminal domain and a C‐terminal domain (Figure 6E). To map the region responsible for its interaction with RNF39, we generated a series of RINT1 truncation MUTs. Our results demonstrate that the C‐terminal domain is both necessary and sufficient for binding to RNF39 (Figure 6F). Key interface residues from both RNF39 and RINT1 were identified, and their spatial proximity suggested a stable protein–protein interaction interface. To further validate this interaction biochemically, GST pulldown assays were performed using bacterially expressed GST‐tagged RNF39 fusion protein and whole‐cell lysates from Flag‐RINT1‐overexpressing cells. RINT1 was specifically retained by GST‐RNF39 but not by GST alone, confirming a direct physical interaction in vitro (Figure 6G). Together, these computational and experimental approaches support a direct and specific interaction between RNF39 and RINT1 in COAD cells, providing a foundation for further mechanistic studies.

### 3.7 RNF39 promotes ubiquitin‐mediated degradation of RINT1 in colorectal cancer cells

**FIGURE 7:**
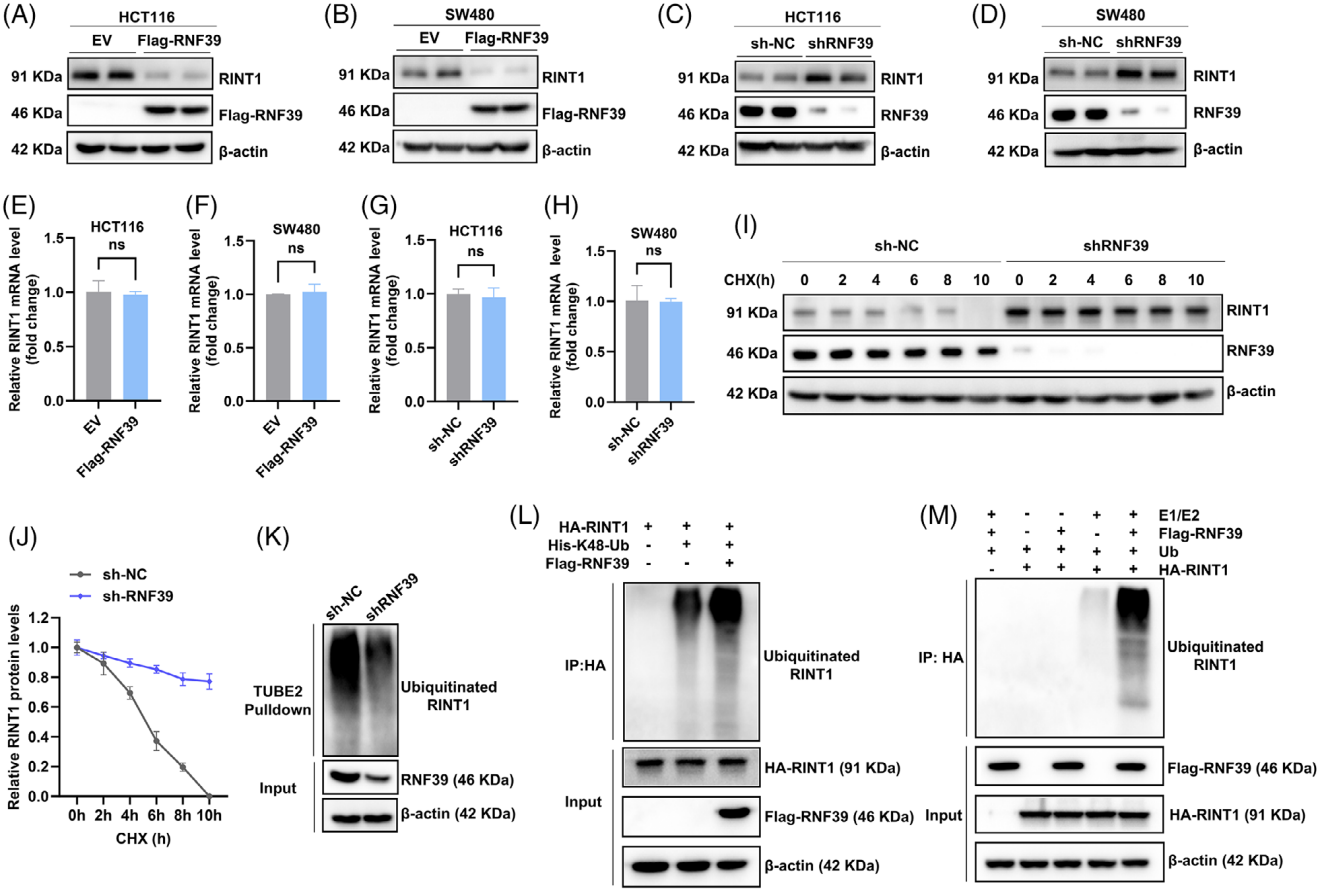
RNF39 promotes ubiquitin‐dependent degradation of RINT1 in colorectal adenocarcinoma cells. (A, B) Western blot analysis of RINT1 protein levels in HCT116 (A) and SW480 (B) cells following RNF39 overexpression. (C, D) Western blot showing increased RINT1 protein levels upon RNF39 knockdown (shRNF39) in HCT116 (C) and SW480 (D) cells. (E–H) qRT‐PCR analysis of RINT1 mRNA levels in RNF39‐overexpressing (E, F) or RNF39‐depleted (G, H) cells, indicating no significant changes. (I) Time‐course western blot analysis of RINT1 protein stability following cycloheximide (CHX) treatment (0–10 h) in SW480 cells with or without RNF39 knockdown. (J) Quantification of RINT1 protein levels over time from (I), normalized to time 0 and plotted as relative intensity. (K) TUBE2 pulldown assay of ubiquitinated RINT1 in control and RNF39‐depleted SW480 cells. (L) Ubiquitination of RINT1 in HEK293T cells. Cells were co‐transfected with HA‐RINT1, Flag‐RNF39 (or vector) and His‐tagged K48‐only ubiquitin (His‐K48‐Ub). HA‐RINT1 was immunoprecipitated, and K48‐linked polyubiquitinated RINT1 was detected by anti‐His immunoblotting. (M) In vitro ubiquitination of RINT1. Lysates from HEK293T cells expressing HA‐RINT1 and/or Flag‐RNF39 were incubated with recombinant E1, E2, ATP and wild‐type ubiquitin. After HA immunoprecipitation, RINT1–ubiquitin conjugates were detected by anti‐HA immunoblotting and were only evident when both RNF39 and E1/E2 were present.

To elucidate the functional consequences of the RNF39–RINT1 interaction, we investigated whether RNF39 regulates RINT1 protein stability via the ubiquitin–proteasome pathway. RNF39 overexpression in HCT116 and SW480 cells resulted in a marked reduction in endogenous RINT1 protein levels, as shown by immunoblotting (Figure 7A,B). Conversely, RNF39 knockdown via shRNA led to a substantial accumulation of RINT1 protein in both cell lines (Figure 7C,D), suggesting that RNF39 may facilitate RINT1 degradation. To determine whether RNF39 regulates RINT1 at the transcriptional or post‐transcriptional level, we examined RINT1 mRNA levels in both RNF39‐overexpressing and RNF39‐silenced cells. Quantitative RT‐PCR showed no significant differences in RINT1 transcript levels across conditions, indicating that RNF39 does not influence RINT1 expression at the mRNA level (Figure 7E–H). To evaluate the stability of the RINT1 protein, a cycloheximide chase assay was conducted. In RNF39‐depleted SW480 cells, RINT1 protein exhibited prolonged stability compared to control cells, suggesting that RNF39 promotes RINT1 turnover (Figure 7I). Densitometric analysis over time further confirmed this delayed degradation in the absence of RNF39 (Figure 7J). To test whether RNF39 promotes RINT1 degradation through ubiquitination, we conducted tandem ubiquitin‐binding entity (TUBE2)‐based pulldown assays. The results revealed a reduction in ubiquitinated RINT1 species in RNF39‐knockdown cells, consistent with impaired ubiquitin conjugation (Figure 7K). Co‐expression of Flag‐RNF39 in HEK293T cells, along with HA‐RINT1 and His‐K48‐only ubiquitin, robustly promoted the K48‐linked polyubiquitination of RINT1 (Figure 7L). To further demonstrate that RNF39 directly catalyses RINT1 ubiquitination, we next performed an in vitro ubiquitination assay using HA‐RINT1– and Flag‐RNF39–expressing cell lysates together with recombinant E1, E2 and WT ubiquitin. In this reconstituted system, RINT1–ubiquitin conjugates were robustly detected only when both RNF39 and the E1/E2 enzymatic cascade were present (Figure 7M). Together, these results support the conclusion that RINT1 is a direct substrate of RNF39‐mediated ubiquitination and proteasomal degradation in our colorectal cancer models.

### 3.8 Loss of RNF39 sensitizes colorectal cancer cells to ER stress by enhancing UPR activation and apoptosis

**FIGURE 8:**
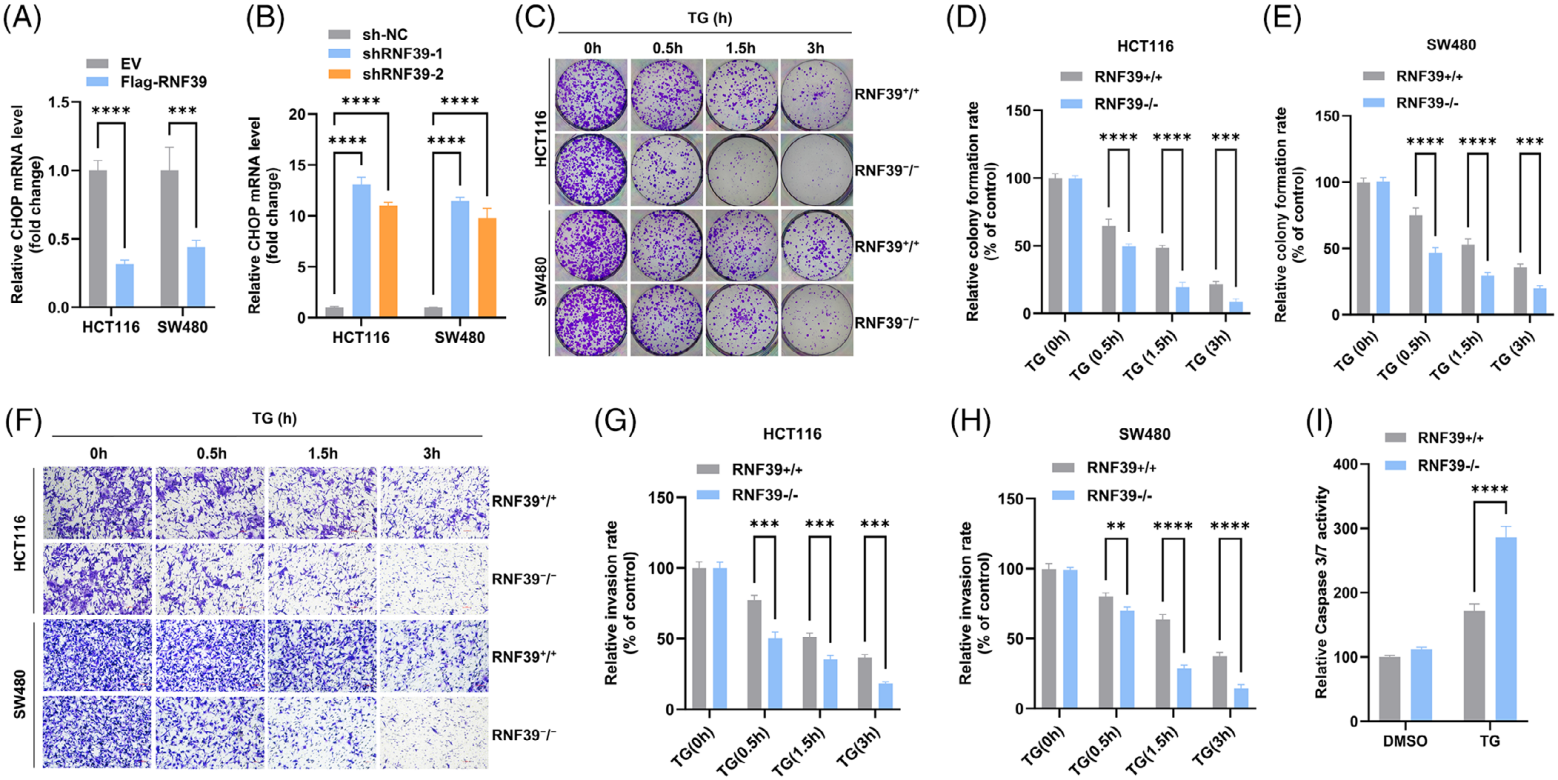
RNF39 modulates cellular sensitivity to ER stress and UPR‐mediated apoptosis in colorectal cancer cells. (A, B) qRT‐PCR analysis of CHOP mRNA levels in HCT116 and SW480 cells following RNF39 overexpression (A) or knockdown (B). (C) Representative images of colony formation assays in RNF39⁺/⁺ and RNF39^−^/^−^ HCT116 and SW480 cells treated with thapsigargin (TG) for the indicated time points (0,.5, 1.5, 3 h). (D, E) Quantification of relative colony numbers in HCT116 (D) and SW480 (E) cells after TG exposure. (F) Representative images of transwell invasion assays performed on RNF39⁺/⁺ and RNF39^−^/^−^ cells treated with TG for 0–3 h. (G, H) Quantification of invaded cells in HCT116 (G) and SW480 (H) cells under the indicated TG treatment durations. (I) Caspase‐3/7 activity assay in RNF39⁺/⁺ and RNF39^−^/^−^ cells treated with TG for 3 h. Data represent mean ± SD of triplicates.

Given the role of RNF39 in targeting RINT1 for proteasomal degradation and the implication of RINT1 in ER homeostasis,^26^ we investigated whether RNF39 regulates the UPR and cellular adaptation under ER stress. The UPR effector CHOP, a pro‐apoptotic transcription factor activated during sustained ER stress,^8^ was assessed as a downstream readout. Overexpression of RNF39 in HCT116 and SW480 cells led to a significant reduction in CHOP mRNA levels (Figure 8A), whereas RNF39 knockdown induced a robust increase in CHOP expression (Figure 8B), indicating that RNF39 negatively regulates CHOP under basal conditions. To determine the impact of RNF39 on cell survival under ER stress, we compared the sensitivity of isogenic WT (RNF39⁺/⁺) and knockout (RNF39^−^/^−^) cells to thapsigargin (TG), a SERCA inhibitor that disrupts Ca^2^⁺ homeostasis.^5^ Colony formation assays revealed a dose‐ and time‐dependent suppression of clonogenic capacity following TG treatment, with RNF39^−^/^−^ cells exhibiting a significantly greater reduction in colony numbers compared to their WT counterparts (Figure 8C–E). In parallel, transwell invasion assays demonstrated that RNF39^−^/^−^ cells were more sensitive to TG‐induced suppression of invasion (Figure 8F–H), suggesting a heightened stress vulnerability in the absence of RNF39. To determine whether RNF39 deficiency promotes apoptosis under ER stress, caspase‐3/7 activity assays were performed in TG‐treated RNF39⁺/⁺ and RNF39^−^/^−^ cells. Caspase‐3/7 activity was significantly elevated in RNF39‐deficient cells compared to WT controls (Figure 8I), consistent with enhanced apoptotic signalling. These results support a model in which RNF39 attenuates ER stress‐induced apoptosis by modulating UPR output, in part through regulation of RINT1 stability and CHOP expression.

### 3.9 RNF39 promotes colorectal tumour progression and modulates ER stress responses through RINT1 degradation

**FIGURE 9:**
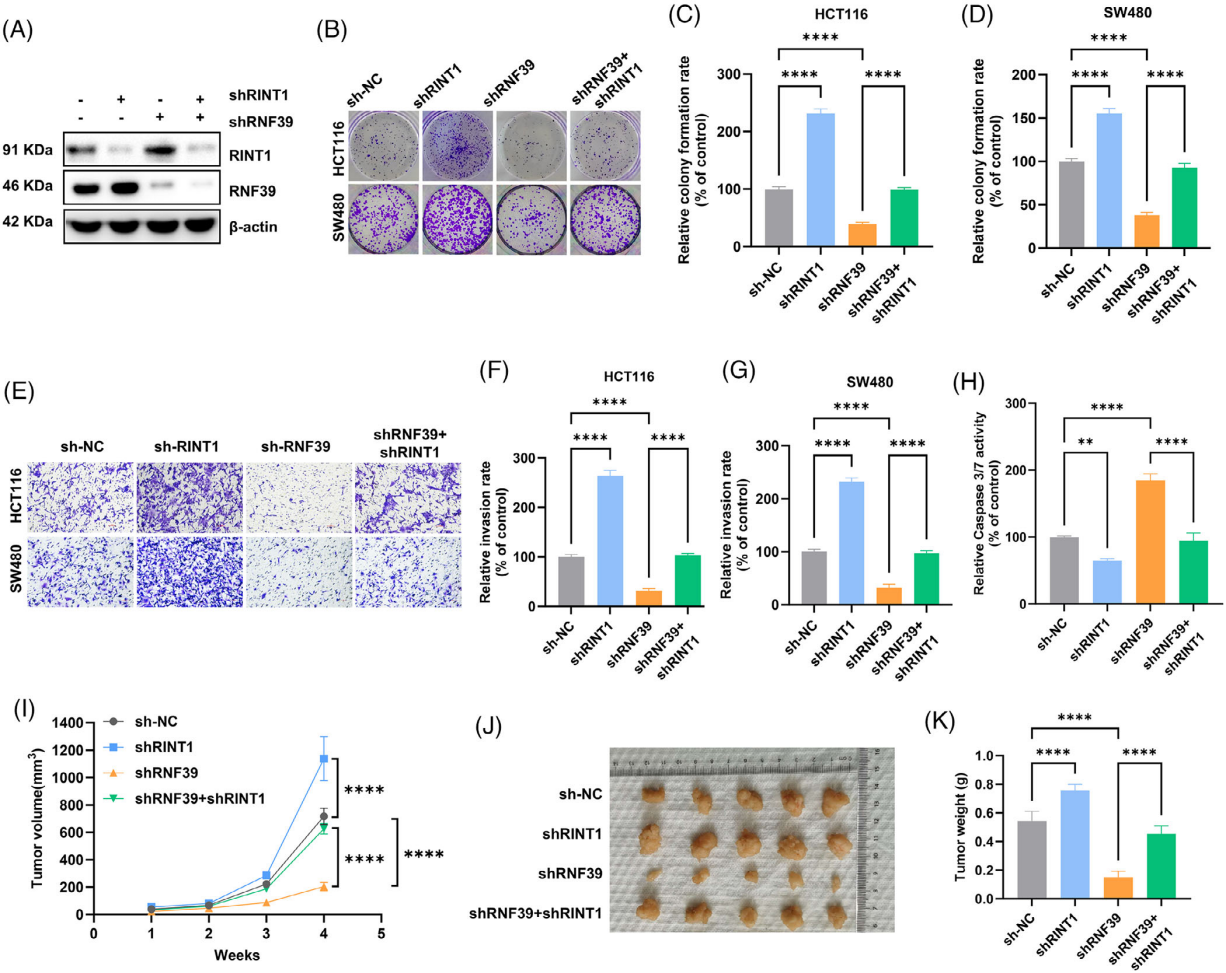
RNF39 modulates colorectal cancer progression and ER stress responses through RINT1 degradation. (A) Immunoblot validation of RNF39 and RINT1 knockdown in HCT116 and SW480 cells transduced with sh‐NC, shRNF39, shRINT1 or shRNF39+shRINT1. (B) Representative images of colony formation assays across the four groups. (C, D) Quantification of colony‐forming ability in HCT116 (C) and SW480 (D) cells. (E) Representative images from transwell invasion assays in each group. (F, G) Quantification of invaded cells in HCT116 (F) and SW480 (G) cells. (H) Caspase‐3/7 activity assay in SW480 cells, comparing apoptotic response across groups. (I) Tumour growth curves from xenograft experiments using stably transduced HCT116 cells implanted into BALB/c nude mice (*n* = 5 per group). (J) Representative images of excised tumours at day 28. (K) Tumour weights at endpoint across the four experimental conditions. Data are presented as mean ± SD; *p*‐values were determined by one‐way ANOVA with post‐hoc testing.

To determine whether RINT1 degradation is a critical mediator of RNF39's oncogenic functions, we generated cells stably expressing control shRNA, shRNA targeting RNF39, shRNA targeting RINT1 or co‐expressing shRNAs for both RNF39 and RINT1. Immunoblot analysis confirmed effective knockdown of RNF39 and RINT1 across the relevant groups (Figure 9A). Functionally, RNF39 knockdown significantly suppressed clonogenic growth in both HCT116 and SW480 cells, whereas RINT1 knockdown alone moderately enhanced colony formation. Importantly, co‐silencing of RINT1 in RNF39‐depleted cells partially rescued the growth‐suppressive effects of RNF39 loss (Figure 9B–D). Similar trends were observed in transwell invasion assays, where RNF39 silencing reduced invasive capacity, and this phenotype was partially reversed by concurrent RINT1 depletion (Figure 9E–G). These findings identify RINT1 as a key downstream effector through which RNF39 promotes tumour cell proliferation and invasion. To assess the impact on apoptotic signalling, we measured caspase‐3/7 activity. RNF39 silencing led to increased caspase‐3/7 activation, consistent with enhanced apoptosis, while simultaneous knockdown of RINT1 significantly reduced caspase activity in RNF39‐depleted cells (Figure 9H). These results suggest that the pro‐apoptotic phenotype induced by RNF39 loss is at least partially dependent on RINT1 accumulation. To validate these observations in vivo, stably modified HCT116 cells were subcutaneously implanted into immunodeficient nude mice. Tumour volume measurements over 4 weeks revealed that RNF39 silencing significantly suppressed tumour growth, while co‐silencing of RINT1 with RNF39 restored tumorigenic potential (Figure 9I). Tumour mass at endpoint was also significantly reduced in the RNF39 knockdown group, and this reduction was reversed upon RINT1 depletion (Figure 9J,K). These findings collectively indicate that RNF39 facilitates colorectal cancer progression and suppresses ER stress‐induced apoptosis by targeting RINT1 for degradation. RINT1 acts as a functional downstream effector of RNF39, integrating proteostasis regulation with tumour growth and survival signalling.

### 3.10 Schematic model of the RNF39–RINT1 regulatory axis in colorectal cancer

**FIGURE 10:**
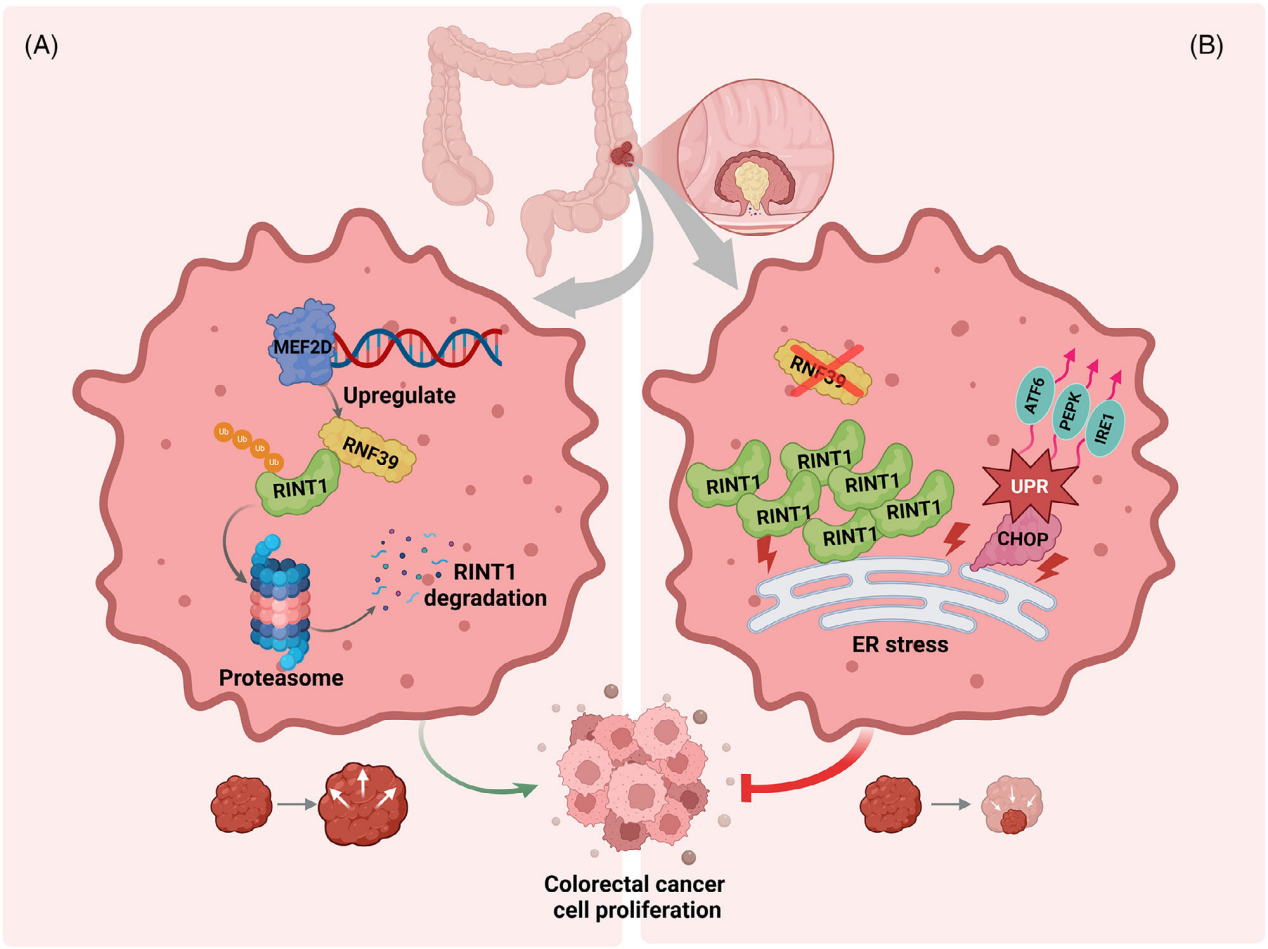
Schematic model of the RNF39–RINT1 regulatory axis in colorectal cancer. (A) In colorectal cancer cells, MEF2D transcriptionally activates RNF39, which functions as an E3 ubiquitin ligase targeting RINT1 for polyubiquitination and proteasomal degradation. Reduced RINT1 levels maintain ER homeostasis and promote tumour cell proliferation. (B) Loss of RNF39 results in RINT1 protein accumulation, leading to ER stress and activation of the unfolded protein response (UPR) via PERK, ATF6 and IRE1 signalling pathways. Upregulation of CHOP promotes pro‐apoptotic signalling, limiting colorectal cancer cell growth. This schematic summarizes a proposed working model rather than a fully delineated pathway.

To provide an integrated mechanistic view of our findings, we developed a schematic model summarizing the molecular pathway by which RNF39 regulates colorectal cancer cell proliferation via RINT1‐mediated ER stress responses. RNF39 was previously linked to immune signalling but is co‐opted by tumour cells to regulate ER stress and survival in COAD. As shown in Figure 10A, the transcription factor MEF2D upregulates RNF39 expression, which in turn promotes the polyubiquitination and proteasomal degradation of RINT1. Depletion of RINT1 facilitates ER homeostasis and supports colorectal cancer cell growth by limiting activation of the UPR. In contrast, RNF39 deficiency leads to accumulation of RINT1 protein (Figure 10B), which disrupts ER proteostasis and triggers UPR signalling through canonical effectors including PERK, ATF6 and IRE1. Sustained UPR activation enhances CHOP expression and promotes apoptotic pathways, thereby suppressing tumour cell proliferation and invasion. This model underscores a functional axis − MEF2D–RNF39–RINT1 − that connects transcriptional regulation, proteasomal degradation, ER stress adaptation and colorectal cancer progression. While the data support a functional connection among MEF2D, RNF39 and RINT1, this model represents a working hypothesis derived from correlative and experimental evidence rather than a fully established pathway.

## 4 DISCUSSION

In this study, we identify RNF39 as a previously uncharacterized RING‐type E3 ubiquitin ligase that drives colorectal cancer progression by promoting ubiquitin–proteasome–mediated degradation of RINT1 and dampening ER stress–induced apoptosis. Integrated analyses of TCGA‐COAD and multiple GEO cohorts reveal that RNF39 is consistently upregulated in colorectal tumours and correlates with adverse clinicopathologic features and poor prognosis; these convergent transcriptomic observations, together with immunohistochemical validation, provide the rationale to prioritize RNF39 for mechanistic interrogation among RING E3 ligases. Functionally, loss‐of‐function perturbation of RNF39 in colorectal cancer cells profoundly attenuated proliferative capacity, invasive behaviour and xenograft tumour growth, whereas enforced RNF39 expression reinforced these malignant traits. This pro‐oncogenic profile stands in sharp contrast to that of RING ligases such as RNF43, which has been reported to exert tumour‐suppressive effects in colorectal cancer,^13^, ^14^ underscoring substantial functional divergence within the RING E3 family and supporting RNF39 as an active driver of colorectal tumour progression rather than merely a correlative biomarker.

Although RNF39 has long been annotated as an RNF protein and putative E3 ligase since the 1990s, its biological role in tumorigenesis, particularly in epithelial malignancies such as COAD, has remained undefined. Our study fills this gap by revealing its function in RINT1 degradation and ER stress regulation. These findings prompted us to investigate the upstream regulatory mechanisms controlling RNF39 expression. Our data demonstrate that the transcription factor MEF2D serves as a key upstream regulator of RNF39. MEF2D expression is positively correlated with RNF39 across patient samples, and its overexpression is sufficient to induce RNF39 transcription. This transcriptional link integrates RNF39 into a broader oncogenic regulatory network, where MEF2D acts as a nodal point coordinating transcriptional and post‐translational pathways that support tumour cell survival. Given that MEF2D is frequently activated in stress‐adaptive transcriptional programmes, its regulation of RNF39 places this E3 ligase under the control of oncogenic signalling networks that integrate metabolic and proteostatic responses.

Functional studies in vitro and in vivo confirmed the pro‐tumorigenic role of RNF39. This phenotype contrasts with the loss‐of‐function behaviour observed for RNF43 or RNF43 mutations, underscoring that distinct RNF ligases can exert opposite influences on colorectal tumorigenesis.^13^, ^14^ Silencing of RNF39 significantly suppressed colorectal cancer cell proliferation, invasion and tumour growth in xenograft models, while ectopic RNF39 expression enhanced these malignant phenotypes. These observations prompted the search for RNF39‐interacting partners, leading to the identification of RINT1 as a direct substrate. RINT1, a known regulator of ER and Golgi integrity,^25^, ^26^ is targeted by RNF39 for proteasomal degradation through K48‐linked ubiquitination. This post‐translational regulation is functionally critical, as RINT1 accumulation upon RNF39 loss leads to sustained activation of the UPR, induction of CHOP and increased susceptibility to ER stress‐induced apoptosis. Although K48‐linked ubiquitination appears to be the predominant mode of RINT1 modification in our system, other ubiquitin chain types and the precise lysine residues targeted by RNF39 remain to be defined and will be addressed in future proteomic, mass spectrometry–based analyses.

Importantly, genetic rescue experiments revealed that co‐depletion of RINT1 in RNF39‐deficient cells partially restored proliferative and invasive capacities and attenuated apoptotic signalling. This observation implies that RNF39‐driven proteasomal degradation of RINT1 acts as a molecular switch controlling the balance between survival and apoptosis under ER stress. These results establish that the oncogenic function of RNF39 is mediated, at least in part, through RINT1 degradation and suppression of ER stress responses. Our findings thus define a novel MEF2D–RNF39–RINT1–UPR signalling axis that couples transcriptional activation and proteostasis regulation to tumour progression in COAD.

While this study provides a mechanistic framework connecting RNF39 to ER stress modulation and colorectal cancer development, several questions remain. Because the C108S mutation abrogates RNF39's ability to promote tumorigenesis, its oncogenic activity clearly depends on its E3 ligase catalytic function, highlighting the ubiquitin–proteasome system as a therapeutic vulnerability. From a translational perspective, RNF39 overexpression correlates with poor prognosis and could serve as a biomarker for stress‐adaptive tumour phenotypes. Inhibition of RNF39 might restore sensitivity to ER stress‐inducing agents, offering a novel therapeutic strategy. First, the precise downstream targets of RINT1 that mediate its pro‐apoptotic effects under ER stress conditions warrant further elucidation. Second, given the complexity of ER stress signalling, it will be important to determine whether RNF39 selectively modulates specific branches of the UPR (e.g. PERK–eIF2α–CHOP vs. ATF6 or IRE1) and how this regulation impacts tumour cell fate decisions. Third, although our findings implicate RNF39 as a potential therapeutic target, its druggability remains unexplored. Targeted degradation of RNF39 or disruption of its interaction with RINT1 could offer a novel therapeutic strategy, but pharmacological validation is needed.

Therapeutically, targeting RNF39 or disrupting its interaction with RINT1 could sensitize colorectal cancer cells to chemotherapy and ER stress–based treatments. Moreover, RNF39 may have broader functional implications beyond COAD. Its consistent upregulation in other cancer types suggests a conserved role in proteostasis and cellular stress adaptation, which merits further investigation. Notably, RNF39 has been implicated in immune regulation through modulation of antiviral and innate immune signalling pathways,^24^, ^33^ and its dual role in proteostasis and immune signalling may contribute to immune evasion mechanisms in colorectal tumours. Given the established link between ER stress, immune modulation and tumour adaptation, RNF39 may represent a molecular bridge integrating stress and immune signalling in cancer. Cross‐cancer analyses could reveal whether RNF39‐driven degradation of RINT1 or other substrates is a generalizable mechanism co‐opted by tumour cells to bypass ER stress‐induced apoptosis. Future studies employing patient‐derived organoids and pharmacologic inhibitors will be essential to validate the clinical utility of targeting RNF39.

In summary, our study establishes RNF39 as a critical mediator of colorectal cancer progression through its regulation of RINT1 stability and ER stress responses. By linking transcriptional control with ubiquitin‐dependent proteolysis, RNF39 enables tumour cells to maintain ER homeostasis and evade stress‐induced apoptosis. These findings not only provide mechanistic insight into tumour adaptation to ER stress but also identify RNF39 as a promising molecular target for therapeutic intervention in colorectal cancer.

## AUTHOR CONTRIBUTIONS

L.C., C.Y., T.Y., K.H., X.L., X.S. and X.J. contributed to data acquisition and analysis. B.L. designed the study. X.L., X.S. and X.J. assisted in report preparation. B.L. and X.J. supervised the study. All authors have read and approved the final manuscript.

## FUNDING INFORMATION

This study was supported by the National Natural Science Foundation of China (82273167), the Jiangsu Province Basic Research Program Natural Science Foundation (Outstanding Youth Fund Project, BK20220063), the Key Program of Basic Science (Natural Science) of Jiangsu Province (22KJA350001), the ‘Huaguo Mountain Talent Plan (Innovative Talents, Bin Liu)’ and the Livelihood Science and Technology Project of Jiuquan Science and Technology Bureau (2024MA1071)

## CONFLICT OF INTEREST S ，TATEMENT

The authors declare no conflicts of interest.

## ETHICAL APPROVAL STATEMENT

All animal experiments were conducted in accordance with the guidelines and regulations set forth by the Experiment Ethics Review Committee of Jiangsu Ocean University and were approved by this committee (Approval No. Jou2025022606). The use of human tissue samples in this study was approved by the Ethics Committee of Ruijin Hospital, Shanghai Jiao Tong University School of Medicine (Approval No. KY2025022). and the Livelihood Science and Technology Project of Jiuquan Science and Technology Bureau (2024MA1071)

## Supporting information



Supporting Information

## Data Availability

The datasets supporting the conclusions of this article are available in the Gene Expression Omnibus repository.
