# Supplementary material for: RNF39 promotes colorectal cancer progression by driving RINT1 degradation and suppressing ER stress‐induced apoptosis
Source: Clin Transl Med. 2025 Dec 28;16(1):e70577. doi: 10.1002/ctm2.70577 (PMC12745170; doi:10.1002/ctm2.70577)
Supplement: Supplementary file 1 — Supporting Information [file CTM2-16-e70577-s001.docx]

# Supplementary Materials

## Primer and RNA Sequences

The following primer sequences were used for quantitative real-time PCR (qRT-PCR), chIP-PCR and shRNA/sgRNA sequences were used for gene knockdown and knockout experiments.

| Type | Target / Name | Sequence (5'→3') | Notes |
| --- | --- | --- | --- |
| qPCR Primer | RNF39-F | AGCCTGAGGTCTAATGTGCG | Forward primer |
| qPCR Primer | RNF39-R | GTTGGGACTTCAAATCGTCTCC | Reverse primer |
| qPCR Primer | RINT1-F | TGAAAGTGGTGACGAAAGGAAG | Forward primer |
| qPCR Primer | RINT1-R | AATGCAGACACATAAGAAGGGAG | Reverse primer |
| qPCR Primer | CHOP-F | GGAAACAGAGTGGTCATTCCC | Forward primer |
| qPCR Primer | CHOP-R | CTGCTTGAGCCGTTCATTCTC | Reverse primer |
| qPCR Primer | GAPDH-F | GAAGGTGAAGGTCGGAGTCA | Forward primer |
| qPCR Primer | GAPDH-R | GGCTGTTGTCATACTTCTCATGG | Reverse primer |
| qPCR Primer | ACTB-F | CATGTACGTTGCTATCCAGGC | Forward primer |
| qPCR Primer | ACTB-R | CTCCTTAATGTCACGCACGAT | Reverse primer |
| shRNA | shMEF2D | CAACAGCCTAAACAAGGTCAT | MEF2D knockdown |
| shRNA | shRNF39-1 | AGGATCTCCCTGAAGATTACC | RNF39 knockdown |
| shRNA | shRNF39-2 | AGGAAGACATGGAGACGATTC | RNF39 knockdown |
| shRNA | shRINT1 | GAAGAGGTTCAGGTATCACTT | RINT1 knockdown |
| shRNA | shCtrl | GAGTTAGCAAGTTCAGCACCT | Non-targeting control |
| sgRNA | sgRNF39 | TCTCGGAGGACTCTCGGCAC | CRISPR targeting RNF39 exon 1 |
| ChIP-PCR Primer | GAPDH-F | CAGGAGGCATTGCTGATGAT | Forward primer |
| ChIP-PCR Primer | GAPDH-R | GAAGGCTGGGGCTCATTT | Reverse primer |
| ChIP-PCR Primer | RNF39-F | AGCCTGAGGTCTAATGTGCG | Forward primer |
| ChIP-PCR Primer | RNF39-R | GTTGGGACTTCAAATCGTCTCC | Reverse primer |

## Antibodies Used in Figures

The following antibodies were used for Western blotting (WB), immunoprecipitation (IP), and other relevant experiments in Figures. Only antibodies with confirmed usage are listed.

| Antibody | Host | Application | Dilution | Supplier / Catalog No. |
| --- | --- | --- | --- | --- |
| RNF39 | Goat | WB, IP | 1:1000 | Santa Cruz Biotechnology, sc-169206 |
| RINT1 | Rabbit | WB, IP | 1:1000 | Sigma-Aldrich, HPA019875 |
| MEF2D | Rabbit | WB | 1:1000 | Affinity, AF7888 |
| HA (Tag) | Rabbit | WB, IP | 1:1000 | Cell Signaling Technology, #3724 |
| Flag (Tag) | Mouse | WB | 1:1000 | Sigma-Aldrich, F1804 |
| His (Tag) | Mouse | WB | 1:1000 | Cell Signaling Technology, #2366 |
| Polyubiquitin (K48-linked) | Rabbit | WB | 1:1000 | Cell Signaling Technology, #8081 |
| Polyubiquitin (pan) | Rabbit | WB | 1:1000 | Enzo Life Sciences, BML-PW8805 |
| HRP anti-Rabbit | Goat | WB | 1:5000 | Jackson ImmunoResearch, 111-035-003 |
| HRP anti-Mouse | Goat | WB | 1:5000 | Jackson ImmunoResearch, 115-035-003 |
| GST(Tag) | Mouse | GST pull-down | 1:500 | Santa Cruz Biotechnology, sc-138 |
